# Cannabis Use, Perspectives, and Experiences Among Patients Receiving Hemodialysis: A Descriptive Patient Survey

**DOI:** 10.1177/20543581241274002

**Published:** 2024-09-21

**Authors:** Josephine Ho, Jennifer Harrison, Marisa Battistella

**Affiliations:** 1 University Health Network, Toronto, ON, Canada; 2 University of Toronto, Toronto, ON, Canada

**Keywords:** cannabis, hemodialysis, kidney disease, survey, symptoms

## Abstract

**Background:**

Patients with chronic kidney disease experience high burden of symptoms, negatively affecting their quality of life. Medication therapy is often initiated to address these symptoms but is limited by variable efficacy and high pill burden. There is interest among clinicians and patients to explore cannabis and cannabinoids as an alternative treatment to manage symptoms related to kidney disease.

**Objective:**

The objectives were to characterize cannabis use among patients receiving maintenance hemodialysis (HD), to describe patient perspectives on cannabis, and to explore patient experiences with their kidney health care team related to cannabis.

**Design:**

This was a descriptive, cross-sectional paper-based patient survey.

**Setting/Participants:**

Patients receiving maintenance HD at Toronto General Hospital in the ambulatory setting between July and August 2020 were included.

**Methods:**

A 33-item questionnaire was developed to address the study questions based on existing cannabis questionnaires and input from kidney specialist physicians, pharmacists, kidney nurse practitioners, and patients. The questionnaire was distributed to patients during their in-center HD session. Patients who chose to participate in the study completed the questionnaire and returned it to the study team.

**Results:**

In total, there were 52 respondents, of which 11 (21%) reported cannabis use in the preceding 3 months, and 23 (44%) reported historical cannabis use. Baseline characteristics were similar between those who used cannabis and those who did not, with a possible trend of cannabis users being younger. The most commonly reported reasons for using cannabis were recreation and symptom management. Those who reported using cannabis for symptom management were doing so without medical authorization or documentation. Common symptoms that cannabis was used to self-treat were insomnia, anxiety, and/or non-neuropathic pain. Dried flower was the most common type of product used, and smoking was the most common route. Care gaps and opportunities to improve patient care related to cannabis use were identified, related to monitoring and management of adverse effects, management of drug interactions, harm reduction strategies, informed decision-making, and prescriber education.

**Limitations:**

The overall participation rate was low, at approximately 17%, possibly related to the COVID-19 pandemic, lack of interest, or fear of revealing cannabis use. Non-response bias is a possible limitation as this was a voluntary survey. The questionnaire was limited to multiple-choice and Likert scale questions, therefore limiting the depth of patient responses.

**Conclusions:**

Our study showed that cannabis use among patients receiving HD is common and comparable with the general population. Patients may be using cannabis to self-manage symptoms related to kidney disease, without the involvement of the health care team. Multiple opportunities to improve patient care related to cannabis use were identified.

## Abrege

**Contexte:**

Les symptômes liés à l’insuffisance rénale représentent un lourd fardeau pour les patients qui en sont atteints, ce qui affecte négativement leur qualité de vie. Un traitement médicamenteux est souvent prescrit pour soulager les symptômes, mais il est limité par son efficacité variable et le nombre élevé de médicaments. Les patients et les cliniciens sont ouverts à explorer d’autres avenues pour gérer les symptômes de l’insuffisance rénale, notamment la consommation de cannabis et de cannabinoïdes.

**Objectifs:**

Caractériser la consommation de cannabis chez les patients recevant des traitements d’hémodialyse (HD) chronique; décrire les perspectives des patients sur le cannabis et examiner les expériences des patients avec leurs équipes de soins en lien avec la consommation de cannabis.

**Type d’étude:**

Enquête transversale descriptive sous forme d’un questionnaire papier remis aux patients.

**Cadre et sujets de l’étude:**

Les patients qui suivaient des traitements d’HD chronique en ambulatoire à l’Hôpital général de Toronto en juillet et août 2020.

**Méthodologie:**

Pour répondre aux objectifs de l’étude, un questionnaire en 33 points a été élaboré à partir de questionnaires existants sur la consommation de cannabis et des commentaires de néphrologues, pharmaciens, infirmières praticiennes en néphrologie et patients. Le questionnaire a été distribué aux patients pendant une séance d’HD en centre. Les personnes qui ont choisi de participer à l’étude l’ont rempli et l’ont renvoyé à l’équipe de recherche.

**Résultats:**

En tout, 52 personnes ont répondu au questionnaire, desquelles 11 (21%) ont déclaré avoir consommé du cannabis dans les trois mois précédents et 23 (44%) en avoir consommé dans le passé. Les caractéristiques initiales des consommateurs de cannabis étaient semblables à celles des personnes qui n’en consommaient pas; les consommateurs de cannabis ayant tendance à être plus jeunes. On consommait principalement du cannabis pour le côté récréatif et pour gérer les symptômes. Les personnes qui consommaient du cannabis pour gérer leurs symptômes le faisaient sans documentation ou indication médicale. Le cannabis était consommé pour l’auto-traitement de symptômes courants comme l’insomnie, l’anxiété et/ou la douleur non neurogène. Le plus souvent, le cannabis était fumé, et la fleur séchée était le produit le plus utilisé. Des lacunes dans les soins et des occasions d’améliorer les soins aux patients ont été identifiées en lien avec la consommation de cannabis, celles-ci concernaient la surveillance et la gestion des effets indésirables, la gestion des interactions médicamenteuses, les stratégies de réduction des risques, la prise de décisions éclairées et l’éducation des prescripteurs.

**Limites:**

Le taux de participation global a été faible (environ 17%), possiblement en raison de la pandémie de COVID-19, d’un manque d’intérêt ou de la réticence à révéler la consommation de cannabis. La participation était volontaire, un biais de non-réponse est donc possible. Le questionnaire n’était constitué que de questions à choix multiples et à échelle de Likert, ce qui limite la profondeur des réponses.

**Conclusion:**

Notre étude montre que la consommation de cannabis chez les patients sous HD est courante et comparable à celle de la population générale. Les patients peuvent consommer du cannabis pour soulager les symptômes liés à l’insuffisance rénale sans intervention de l’équipe de soins. Le sondage a permis d’identifier plusieurs occasions d’améliorer les soins aux patients en lien avec la consommation de cannabis.

## Introduction

Patients with end-stage kidney disease (ESKD) experience high symptom burden that negatively affects their quality of life. One study showed that those with chronic kidney disease (CKD) stage 4 and above experience an average of 13 undesirable symptoms.^1^ Among patients receiving hemodialysis (HD), commonly reported symptoms include fatigue, pain, decreased appetite, and xerosis.^1^ There is increasing recognition of the need to adequately manage these symptoms to improve quality of life among HD patients.^2^ While medication therapy is often initiated to address these symptoms, limitations include variable efficacy and high pill burden. One study found that patients receiving HD have a mean daily pill burden of 19 oral medications, among the highest of any chronic disease state.^3^

There has been interest in using cannabis and cannabinoids as alternative treatments to manage symptoms related to ESKD.^4-6^ Cannabis refers to the dried plant, of which the most common species are *Cannabis sativa* and *Cannabis indica*.^7^ In animal and human studies, cannabis exerts a range of effects on various systems due to the broad range of the endocannabinoid system. It contains hundreds of chemical compounds and upward of 104 cannabinoids, which bind to cannabinoid receptors.^8^ Of the cannabinoids, cannabidiol (CBD) and delta-9-tetrahydrocannabinol (THC) are the most frequently described due to their pharmacologic properties. Delta-9-tetrahydrocannabinol is commonly referred to as the psychoactive cannabinoid as it may cause a range of effects on emotion and cognition, including euphoria, dysphoria, and hallucinations.^8,9^ Cannabidiol does not have these psychoactive properties and may attenuate some of the undesired effects of THC.^8^

The rationale for exploring cannabis as a treatment alternative in this population includes its efficacy in managing similar symptoms in non-ESKD populations,^10-15^ its lack of dependence on kidney clearance and nephrotoxicity,^4,5^ and recent legislative changes in Canada that have expanded public access. The Cannabis Act legalized cannabis for non-medical purposes in Canada in 2018.^16^ This change in legislation has increased the number of legal venues to access cannabis and also the public interest in cannabis. Use of cannabis for self-management of medical conditions or adverse symptoms is apparent, with the 2023 Canadian Cannabis Survey reporting that 10% of respondents used cannabis for a medical reason in the preceding 12 months, and only 18% reported having medical documentation, suggesting self-directed use.^17^

There is limited published information describing the use of cannabis among HD patients. In 2020, Samaha et al published a single center survey study of 192 patients with CKD (72% receiving HD, 26% receiving peritoneal dialysis, 2% hybrid of both) that focused on assessing restless leg syndrome (RLS) and uremic pruritus, and the use of cannabis to manage these symptoms. They found that 14 of 86 patients with RLS and 5 of 29 patients with uremic pruritus, respectively, reported using cannabis for symptom management.^18^ In 2023, Collister et al published a multi-center survey study of 320 patients with CKD (75.8% in-center HD, 5.4% home HD) regarding patient views of cannabis use. They found that 50.2% of respondents used cannabis in their lifetime, mostly smoking (87.5%), edibles (57.5%), and oils (43.1%). Only 10.3% were previously prescribed cannabis by a health care provider, and the most common reasons for non-prescribed cannabis use were for recreation (52.5%), pain (39.4%), and sleep (35.5%).^19^

This study aimed to address the paucity of information regarding use of cannabis in the HD population as well as explore the interactions patients have with their health care providers regarding cannabis. Our study objectives were to characterize cannabis use among patients receiving HD, to describe patient perspectives on cannabis, and to explore patient experiences with their HD health care team related to cannabis.

## Materials and Methods

### Study Design

This study was a descriptive, cross-sectional paper-based patient survey. Participant consent was implied upon return of completed questionnaires to the research team.

### Questionnaire Development

Existing literature regarding cannabis, ESKD, and patients receiving HD were reviewed to inform the content and background of the questionnaire. Other existing patient/public questionnaires about cannabis were reviewed, such as the Canadian National Cannabis Survey and a previous survey about cannabis in the multi-organ transplant population from 2016.^17,20^ A set of questions (multiple-choice and Likert scale rating) were developed with the goal of addressing the study objectives. The questionnaire was reviewed by several expert groups for content, survey logic, structure, and formatting. These expert groups included kidney specialist physicians, pharmacists, kidney nurse practitioners, a patient care coordinator from the HD unit, and patients receiving HD. The patients were identified by the patient care coordinator and they provided feedback about the questionnaire.

### Study Population

All active patients at the Toronto General Hospital (TGH) ambulatory HD clinic from July to August 2020 were invited to complete the survey. Patients were eligible to participate if they were 18 years of age or older, receiving HD, and able to read and respond to a paper questionnaire written in English.

### Sample Size

At the time of data collection (July-August 2020), there were approximately 300 active ambulatory patients receiving maintenance HD at TGH. We estimated that 50% of these patients were eligible for our study based on our inclusion criteria. As a voluntary survey, we estimated that 70 to 100 patients in total would participate.

### Questionnaire Distribution and Collection

Eligible participants were invited to complete the survey by a member of the study team during their in-center HD session. Patients were given a cover letter outlining the purpose of the survey and the questionnaire. As an initial pilot phase, 30 patients were invited to complete the questionnaire. The purpose of the pilot phase was to have a preliminary analysis for overall completeness and to assess whether modifications were needed to the questionnaire. The questionnaire was then distributed to all eligible participants. Completed questionnaires were returned to a secure collection box by the participant, nurse, or a member of the study team.

### Data Collection and Analysis

Responses from the paper questionnaires were manually transcribed to an electronic (Microsoft Excel 2019) data collection form. Descriptive analyses were done for qualitative results. Means, medians, standard deviations, and interquartile ranges were calculated for quantitative data.

## Results

A 4-paged, 33-item questionnaire was developed after review and feedback from 3 clinical pharmacists, 3 kidney specialist doctors, 1 kidney nurse practitioner, 1 patient care coordinator, and 3 patient volunteers (Supplemental Appendix 1). In the initial pilot phase, 30 patients were invited to complete the questionnaire, of whom 9 did so. Upon review by the study team, no issues requiring modification to the questionnaire were identified and all response fields were complete. At the end of the survey period, of the 300 patients invited, 52 (17%) completed and returned a questionnaire. Of these 52 surveys, 67 response fields were incomplete (3% of total fields). All data collected and reported is based on patient self-report.

### Baseline Characteristics

Of the 52 respondents, 11 (21%) used cannabis in the preceding 3 months (referred to as “current cannabis users”) and 23 (44%) reported historical cannabis use. Baseline characteristics are described in Table 1. Among all respondents, 37% were female, 31% were between the ages 55 and 64, and 23% were between the ages 45 and 54. Diabetes (19%), hypertension (17%), and genetic disease (17%) were the most common causes of kidney disease. With respect to number of current medications, 42% were taking 5 to 10 medications and 37% were taking less than 5 medications. Most patients (90%) did not report current tobacco cigarette use.

**Table 1.** Baseline Characteristics.

Characteristic	Cannabis use status—n (%)		
	Overall n = 52 (100)	Current user^a^ n = 11 (21)	Non-user^b^ n = 41 (79)
Gender			
Female	19 (37)	5 (45)	14 (34)
Male	33 (63)	6 (55)	27 (66)
Age group (years)			
18-24	2 (4)	1 (9)	1 (2)
25-34	5 (10)	1 (9)	4 (10)
35-44	5 (10)	3 (27)	2 (5)
45-54	12 (23)	2 (18)	10 (24)
55-64	16 (31)	4 (36)	12 (29)
65-79	7 (13)	0	7 (17)
80 or older	5 (10)	0	5 (12)
Etiology of kidney disease			
Diabetes	10 (19)	0	10 (24)
Hypertension	9 (17)	2 (18)	7 (17)
Autoimmune disease	2 (4)	1 (9)	1 (2)
Glomerulonephritis	6 (12)	1 (9)	5 (12)
Genetic disease	9 (17)	4 (36)	5 (12)
Not sure	9 (17)	2 (18)	7 (17)
Other	12 (23)	2 (18)	10 (24)
Total duration of HD (years)			
Less than 1	9 (17)	2 (18)	7 (17)
1-5	25 (48)	5 (45)	20 (49)
6-10	4 (8)	0	4 (10)
More than 10	13 (25)	3 (27)	10 (24)
Number of current medications			
Less than 5	19 (37)	3 (27)	16 (39)
5-10	22 (42)	6 (55)	16 (39)
11-15	8 (15)	1 (9)	7 (17)
More than 15	2 (4)	1 (9)	1 (2)
Frequency of alcohol use in the last 12 months			
Never	28 (54)	4 (36)	24 (59)
0-1 times per month	9 (17)	3 (27)	6 (15)
2-3 times per month	6 (12)	2 (18)	4 (10)
Once per week	3 (6)	0	3 (7)
2-5 times per week	2 (4)	1 (1)	1 (2)
Daily or almost daily	3 (6)	1 (9)	2 (5)
Current smoking status			
Not smoking	47 (90)	9 (82)	38 (93)
Occasionally	1 (2)	1 (9)	0
Every day	2 (4)	1 (9)	1 (2)

a Current user in the last 3 months.

b Among the group of non-users, 23 respondents indicated previous historic use (more than 3 months ago).

Among current cannabis users, all were under 65 years of age with 36% between the ages 55 and 64, and 27% between the ages 35 and 44. Genetic disease (36%) and hypertension (18%) were the most common causes of kidney disease in this group.

### Cannabis Use Characteristics

**Figure 1.**
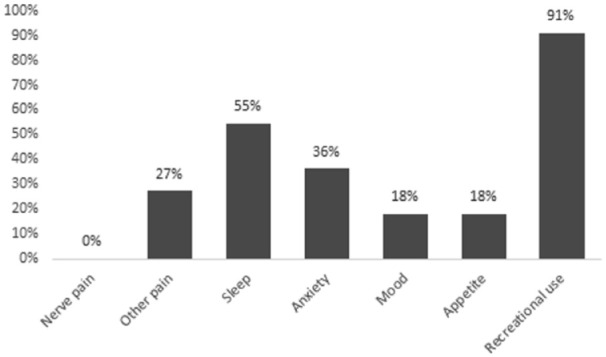
Self-reported reasons for cannabis use among current cannabis users (n = 11).^a^ ^a^Percentages may add up to more than 100% as more than one response was allowed.

**Figure 2.**
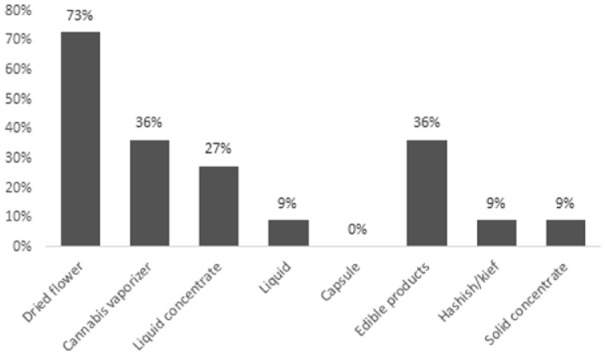
Type(s) of cannabis products used by current cannabis users (n = 11).^a^ ^a^Percentages may add up to more than 100% as more than one response was allowed.

**Figure 3.**
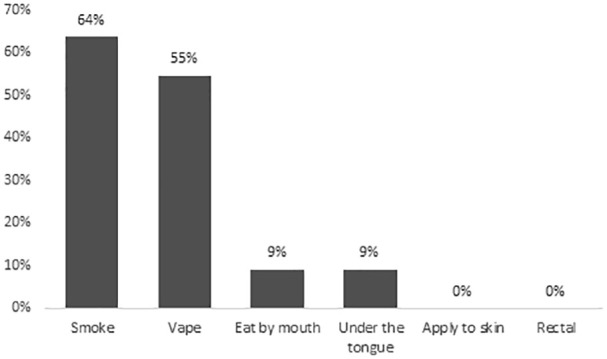
Route of administration of cannabis products reported by current cannabis users in the preceding 3 months (n = 11).^a^ ^a^Percentages may add up to more than 100% as more than one response was allowed.

Among the 11 current cannabis users, the reasons for use were recreation (78%) and/or symptom management (45%). None of the respondents obtained medical authorization or medical documentation for cannabis. The most common symptoms cannabis was used to self-treat were insomnia (55%), anxiety (36%), and/or non-neuropathic pain (27%), as shown in Figure 1. Dried flower was the most common type of cannabis product used (73%), followed by cannabis vaporizers (36%), and/or edible products (36%), as shown in Figure 2. With regards to the relative THC and CBD composition of the cannabis product used, 45% (5 of 11 respondents) did not know, 36% (4 of 11 respondents) used a product with higher THC than CBD, and 18% (2 of 11 respondents) used a product with equal THC and CBD amounts. Smoked (64%) and/or vaporized cannabis (55%) were the most common routes of administration, as shown in Figure 3. Regarding frequency of use, 27% used cannabis at least once daily, and 45% used cannabis once or twice in the preceding 3 months. The most common sources of obtaining cannabis were family and friends (73%) and online licensed producers (27%). Of the 11 respondents, 8 reported the amount of money spent per month to purchase cannabis, with a median of $25 (IQR = $20-138).

### Patient Perspectives on Cannabis

Among all 52 respondents, 50% perceived cannabis to have beneficial health effects, while 25% perceived it to have harmful health effects. Among current cannabis users, 91% reported that cannabis is helpful for symptom management. Specifically, of the 6 patients who used cannabis for sleep, 5 (83%) reported benefit; of the 4 patients who used cannabis for anxiety, 2 (50%) reported benefit; and of the 3 patients who used cannabis for non-neuropathic pain, 2 (67%) reported benefit.

**Figure 4.**
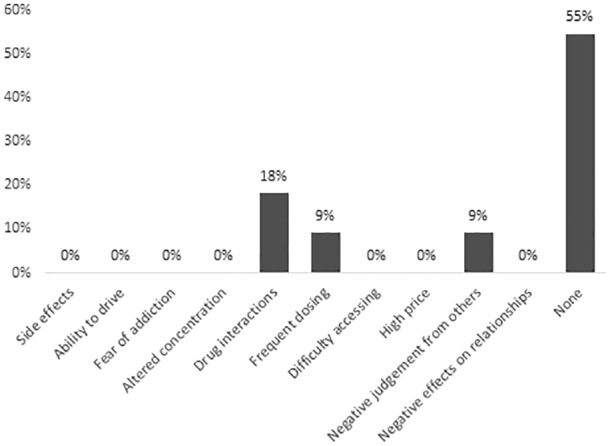
Perceived concerns regarding cannabis among current cannabis users (n = 11).^a^ ^a^Percentages may add up to more than 100% as more than one response was allowed.

Current cannabis users identified perceived benefits as follows: cannabis comes from a natural plant (55%), is more effective than other medications (55%), and is safer than other medications (45%). Among current users, 55% stated they had no concerns about cannabis, 18% were concerned about drug interactions, 9% were concerned about the frequent dosing schedule, and 9% were concerned about negative judgment from others. Other potential harms, including side effects and addiction, were not identified as concerns by any current users (Figure 4).

Among non-users, 39% reported that they had considered using cannabis (29% for medical purposes and 10% for recreational purposes). The most common reasons for not initiating cannabis for those who considered it were concerns about side effects (12%), drug interactions (12%), or that it was not recommended by a physician (12%).

### Patient Experiences Related to Cannabis and the HD Team

**Figure 5.**
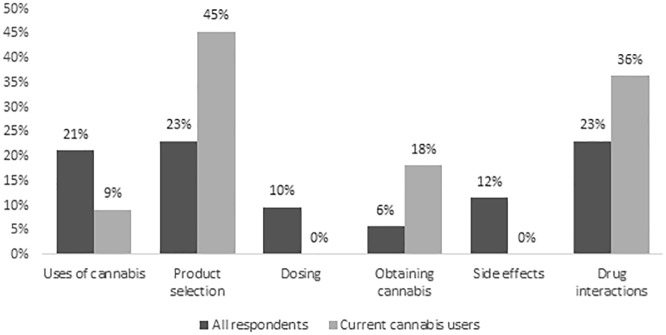
Types of information about cannabis of interest to all respondents (n = 52) and current cannabis users (n = 11).^a^ ^a^Percentages may add up to more than 100% as more than one response was allowed.

Among all respondents, 8% reported being asked by the HD team about cannabis use and 12% reported previously asking the HD team about cannabis. The types of information of interest to respondents were how to select a cannabis product (23%), drug interactions (23%), and/or indications for cannabis use (21%), as shown in Figure 5. Regarding sources of information about cannabis, 21% selected the HD team as being most reliable, followed by medical cannabis clinics (19%), and family physicians (17%).

## Discussion

This study aimed to characterize cannabis use among patients receiving HD, describe patient perspectives related to cannabis use, and evaluate patient experiences related to cannabis and their HD health care team. While other studies have reported on the perspectives of prescribers, nephrologists, and physicians-in-training, this study is one of the first to focus on patient perspectives among those receiving HD.^21-24^

Our study sample appeared to be younger than the Canadian average (mean age of patients with ESKD was reported to be 64 years old)^25^ and compared with a study done by Collister et al^19^ This may have been at least partially due to our survey methodology (ie, patients were required to complete the survey without assistance), which may have unintentionally selected for younger participants. The average age of patients receiving HD at our center was 60 years old, which is younger than the national average, possibly because of our location (downtown urban center).

Our results revealed 3 themes with important patient care and research implications. First, we found that cannabis use is relatively common among our study sample and is often used for symptom management, highlighting its relevance, and a need for clinicians to incorporate a cannabis assessment in the clinical care of patients. In our study, the frequency of cannabis use among patients receiving HD (21%) was comparable with that reported by Collister et al^19^ in a similar but larger survey of cannabis use in patients with CKD (17.8% smoked cannabis, 8.4% used cannabis edibles, and 5.3% used cannabis oils in the last month). Compared with the general population, the 2020 National Cannabis Survey reported that 20% of those over the age of 15 reported cannabis use in the past 3 months.^26^ Notably, we found that 45% were using cannabis for adverse symptom management, but none had formal medical authorization. Collister et al likewise reported that only a small proportion of cannabis users (10.3%) had been prescribed cannabis by a medical practitioner. Furthermore, our study found that only 8% of respondents reported ever being asked about cannabis use by a member of the HD team. This highlights a current lack of clinician involvement in patient self-management of symptoms with cannabis. Potential barriers are insufficient provider knowledge, a lack of routine surveillance of adverse symptoms and cannabis use, and low patient comfort/trust in discussing cannabis in health care settings. A systematic review done by Gardiner et al explored the perspective of health care providers with regards to medical cannabis. They found that although most health care providers showed support for the use of medical cannabis, they reported low self-perceived clinical and legislative knowledge.^21^ In a survey of Canadian nephrologists by Gitau et al,^24^ 59% of respondents felt uncomfortable with their knowledge of medical cannabis literature. Besides addressing this knowledge gap, incorporating questions regarding cannabis use and adverse symptoms in routine patient assessments may facilitate more patient-provider discussions about cannabis use. Provider acknowledgment of cannabis as a therapeutic modality may strengthen patient-clinician relationships by helping patients feel more open to discuss cannabis use.

Next, our study found that the most common symptoms cannabis was being used to manage in our small study sample were insomnia, anxiety, and non-neuropathic pain. However, there is limited evidence to show efficacy or safety of cannabis for these symptoms in patients with kidney disease and/or receiving HD.^4^ In patients without kidney disease, there is some evidence for using cannabis to treat chemotherapy-induced nausea and vomiting (CINV) in the oncology population,^10-12^ for management of neuropathic pain in patients with human immunodeficiency virus (HIV),^13^ and for treatment of anorexia/cachexia in patients with HIV.^14^ In general, most studies excluded patients with kidney disease, and only one small study demonstrated a benefit of a topical cannabinoid in reducing uremic pruritus and xerosis among patients receiving HD.^15^

In non-ESKD populations, there is limited quality evidence for the use of cannabis and cannabinoids in the treatment of anxiety and other mental health conditions. A systematic review done in 2019 by Black et al summarized evidence regarding the use of cannabinoids for the treatment of depression, anxiety, attention deficit hyperactivity disorder, post-traumatic stress disorder, and psychosis. They found that some studies, although low quality, showed that THC with or without CBD improved anxiety symptoms for those with comorbidities, such as chronic non-cancer pain and multiple sclerosis. They did not find any other significant difference of cannabinoids for other mental health conditions, with the exception of one small study showing that THC increased psychosis symptoms.^27^ This lack of data, even in the general population, is an important point to discuss with patients who are interested in using it for improvement of mental health conditions. By identifying the most common symptoms that patients are using cannabis to manage, this may help to focus future research initiatives to these indications in order to inform clinical care.

Finally, we identified high risk cannabis use and opportunities for patient education and harm reduction. The majority of respondents who reported cannabis use either smoked or vaporized cannabis. Similarly, in a survey of patients with CKD, Collister et al^19^ also found that smoked cannabis was the most common route among those who reported using cannabis. This is considered high risk use because cannabis smoke contains many of the same respiratory irritants and toxins as tobacco smoke and is associated with histopathological changes in the respiratory tissue.^8,9^ The 2017 Lower Risk Cannabis Use Guidelines state that smoked cannabis is the most harmful route of using cannabis and they suggest other methods, such as edibles or vaping to avoid deep inhalation of cannabis smoke.^28^ Interestingly, we found that none of the current cannabis users expressed concern for side effects and none expressed any interest in receiving information about side effects. These findings highlight a knowledge gap and an opportunity for the health care team to educate patients about potential risks, discuss lower risk alternatives, and to facilitate informed decision-making among patients. It also emphasizes the need to develop a stronger therapeutic relationship with patients to facilitate patient receptivity to this information.

Another area for harm reduction is in recognizing and managing potential drug interactions with cannabis. This is significant among those receiving HD, due to the numerous concomitant medications for kidney disease and other comorbidities. There are numerous potential drug interactions with significant clinical consequences. Pharmacodynamic interactions with cannabis include central nervous system depressants (increased drowsiness, ataxia), anticholinergic drugs (increased tachycardia, drowsiness, dry mouth), sympathomimetic drugs (increased tachycardia, hypertension), and stimulants (increased tachycardia). Pharmacokinetically, cannabis is metabolized hepatically through cytochrome P450 enzymes (CYP450). Cannabidiol is metabolized mainly by CYP 2C9, 2C19, 3A4, 1A2, and 2D6 enzymes and THC is metabolized mainly by CYP 2C9, 2C19, and 3A4 enzymes.^9^ Therefore, pharmacokinetic interactions with cannabis may occur with inhibitors and inducers of these CYP enzymes, for example, ketoconazole (CYP 3A4 inhibitor) and rifampin (CYP 3A4 inducer). Cannabis itself also inhibits CYP2C9, induces CYP 1A2, and upregulates P-glycoprotein.^9^ Warfarin, a commonly used oral anticoagulant in HD patients, is an example of a drug that has been shown to interact with cannabis. Case reports have described increases in the International Normalized Ratio (INR) among patients taking warfarin and using cannabis (smoked or ingested).^29^ Interestingly, drug interactions were identified as the most common concern among cannabis users in our study. This was also the second most common type of information that respondents identified they would be interested in receiving from the health care team. These findings further underscore the opportunity and need for the health care team to incorporate a discussion of cannabis use into routine clinical care, in order to identify, manage, and educate patients on this topic.

Our study had limitations. First, the overall participation rate was lower than anticipated, at approximately 17% of the total population invited. Although we did not collect data on reasons for non-participation, potential reasons include language barrier, lack of interest, decreased willingness to participate due to the COVID-19 pandemic, and/or fears that revealing information about cannabis use may impact their care. A similar study done by Collister et al achieved a higher response rate (27.3% in person, 9.4% online). Some strategies used in their study that we did not employ for reasons of feasibility include the options of completing surveys by mail or online, including patients from multiple sites, and circulating the survey online (social media, CKD organization websites). Providing assistance from the study team to complete the survey was considered, but not pursued because we wanted to ensure anonymity and mitigate any possible privacy concerns that patients may have had in discussing cannabis use.

As a voluntary survey, non-response bias is another possible factor that may further limit the generalizability of our study. This is a limitation cited in other voluntary surveys as well, such as the Canadian Cannabis Survey.

Finally, for feasibility reasons, this survey consisted of only multiple-choice and Likert scale rating questions, therefore limiting the breadth and depth of responses provided. For future studies, patient and provider interviews may provide more detailed insights and identify other themes.

## Conclusion

In summary, our study found that despite a lack of evidence to support the use of cannabis in kidney disease-related symptom management, a significant proportion of our study population are already using or have considered using cannabis. We identified clear care gaps and multiple opportunities for the health care team to engage with patients on this relevant topic. These opportunities include implementation of routine assessment and discussion around cannabis use; monitoring and management of adverse effects; identification, monitoring, and management of potential drug interactions; as well as provision of information and support for harm reduction strategies and informed decision-making. Clinician acknowledgement that patients now have cannabis legally available to them as a therapeutic modality for kidney disease-related adverse symptom management and engagement with them on this topic may reduce the potential risks associated with cannabis use, improve patient experience, and strengthen therapeutic relationships between patients and their health care team. These approaches will require increased clinician awareness, additional knowledge and training, as well as changes to routine care practices to ensure that providers are able to offer such support in an effective, sensitive, and patient-centric manner.

## Supplemental Material

sj-docx-1-cjk-10.1177_20543581241274002 – Supplemental material for Cannabis Use, Perspectives, and Experiences Among Patients Receiving Hemodialysis: A Descriptive Patient Survey Supplemental material, sj-docx-1-cjk-10.1177_20543581241274002 for Cannabis Use, Perspectives, and Experiences Among Patients Receiving Hemodialysis: A Descriptive Patient Survey by Josephine Ho, Jennifer Harrison and Marisa Battistella in Canadian Journal of Kidney Health and Disease
