# Supplementary material for: Cannabis Use, Perspectives, and Experiences Among Patients Receiving Hemodialysis: A Descriptive Patient Survey
Source: Can J Kidney Health Dis. 2024 Sep 21;11:20543581241274002. doi: 10.1177/20543581241274002 (PMC11418350; doi:10.1177/20543581241274002)
Supplement: sj-docx-1-cjk-10.1177_20543581241274002 – Supplemental material for Cannabis Use, Perspectives, and Experiences Among Patients Receiving Hemodialysis: A Descriptive Patient Survey [file sj-docx-1-cjk-10.1177_20543581241274002.docx]

**Appendix 1 – Patient Questionnaire**

**
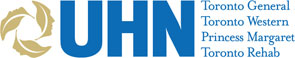
Anonymous Survey on Cannabis in Hemodialysis**

There are 3 sections in this survey and a total of 33 questions. The information collected in this survey is **anonymous** and your answers will not be linked to you. This survey is for patients of the **Toronto General Hospital In-Centre Hemodialysis Unit**.

If you have completed a survey on a previous visit, please do not fill out another one.

**INSTRUCTIONS:**

- Please put an **X** in the circle that corresponds to your answers.
- There are no right or wrong answers – please answer all questions to the best of your ability.
- **Once completed, please return the survey to the Survey Collection Box at the entrance of the hemodialysis units.**

Thank you for your participation.

| **Section 1: Information About You** | | | | | | | | | | | | | | | | | | | | | | | | | | | | | | | | | | | | | | | | | | | | | | | | | | | | | | | | | | | | | | | |  |
| --- | --- | --- | --- | --- | --- | --- | --- | --- | --- | --- | --- | --- | --- | --- | --- | --- | --- | --- | --- | --- | --- | --- | --- | --- | --- | --- | --- | --- | --- | --- | --- | --- | --- | --- | --- | --- | --- | --- | --- | --- | --- | --- | --- | --- | --- | --- | --- | --- | --- | --- | --- | --- | --- | --- | --- | --- | --- | --- | --- | --- | --- | --- | --- | --- |
| 1. **What is your gender?** | | | | | | | | | | | | | | | | | | | | | | | | | | | | | | | | | | | | | | | | | | | | | | | | | | | | | | | | | | | | | | | |  |
|  | | ⃝ Male | | | ⃝ Female | | | | | | | | | | | | ⃝ Other – specify: _______________ | | | | | | | | | | | | | | | | | | | | | | | | | | | | | | | | | | | | | | | | | | | | |  | |  |
| 1. **What is your age group?** | | | | | | | | | | | | | | | | | | | | | | | | | | | | | | | | | | | | | | | | | | | | | | | | | | | | | | | | | | | | | | | |  |
|  | | ⃝ 18-24 | | | ⃝ 25-34 | | | | | | | | | | | | ⃝ 35-44 | | | | | | | | | | | | | | | | | ⃝ 45-54 | | | | | | | | | | | | | | | | ⃝ 55-64 | | | | | | | | | | | |  | |  |
|  | | ⃝ 65-79 | | | ⃝ 80 or older | | | | | | | | | | | | | | | | | | | | | | | | | | | | | | | | | | | | | |  | | | | | | | | | | | | | | |  | | | |  | |  |
| 1. **What is your highest level of education?** | | | | | | | | | | | | | | | | | | | | | | | | | | | | | | | | | | | | | | | | | | | | | | | | | | | | | | | | | | | | | | | |  |
|  | | ⃝ High school diploma | | | | | | | | | | | | | | | | ⃝ Trade certificate or diploma | | | | | | | | | | | | | | | | | | | | | | | | | | | | | | | | | ⃝ College diploma | | | | | | | | | | | | |  |
|  | | ⃝ University degree | | | | | | | | | | | | | | | ⃝ University graduate degree | | | | | | | | | | | | | | | | | | | | | | | | | | | | | | | | | | | | | | | | | | | | | | |  |
|  | | ⃝ Other – specify: __________________________ | | | | | | | | | | | | | | | | | | | | | | | | | | | | | | | | | | | | | | | | | | | | | | | | | | | | | | | | | | | | | |  |
| 1. **How many years (in total) have you been receiving hemodialysis treatment?** | | | | | | | | | | | | | | | | | | | | | | | | | | | | | | | | | | | | | | | | | | | | | | | | | | | | | | | | | | | | | | | |  |
|  | | ⃝ Less than 1 year | | | | | | ⃝ 1-5 years | | | | | | | | | | | | | | | | | | ⃝ 6-10 years | | | | | | | | | | | | | | | ⃝ More than 10 years | | | | | | | | | | | | | | | | | | | | | | |  |
| 1. **Are you on the waiting list for a kidney transplant?** | | | | | | | | | | | | | | | | | | | | | | | | | | | | | | | | | | | | | | | | | | | | | | | | | | | | | | | | | | | | | | | |  |
|  | | ⃝ Yes | | | ⃝ No | | | | | | | | | | | |  | | | | | | | | | | | | | | | | | | | | | | | | | |  | | | | | | | | | | | | | | |  | | | | | |  |
| 1. **What is the cause of your kidney disease?** | | | | | | | | | | | | | | | | | | | | | | | | | | | | | | | | | | | | | | | | | | | | | | | | | | | | | | | | | | | | | | | |  |
|  | | ⃝ Diabetes | | | | | | | | | | | ⃝ Hypertension | | | | | | | | | | | | | | | | | | | | | | | | | | ⃝ Autoimmune disease (eg. lupus) | | | | | | | | | | | | | | | | | | | | | | | | |  |
|  | | ⃝ Glomerulonephritis | | | | | | | | | | | ⃝ Genetic disease (eg. polycystic kidney disease | | | | | | | | | | | | | | | | | | | | | | | | | | | | | | | | | | | | | | | | | | | | | | | | | | |  |
|  | | ⃝ I am not sure | | | | | | | | | | | ⃝ Other – Specify: ________________________ | | | | | | | | | | | | | | | | | | | | | | | | | | | | | | | | | | | | | | | | | | | | | | | | | | |  |
| 1. **How many different medications do you take per day?** | | | | | | | | | | | | | | | | | | | | | | | | | | | | | | | | | | | | | | | | | | | | | | | | | | | | | | | | | | | | | | | |  |
|  | | ⃝ Less than 5 | | | | | | | ⃝ 5-10 | | | | | | | | | | | | | | | | | | | | | | | | ⃝ 11-15 | | | | | | | | | | | | | | | | | | | | | | ⃝ More than 15 | | | | | | | | |  |
|  | | | | | | | | | | | | | | | | | | | | | | | | | | | | | | | | | | | | | | | | | | | | | | | | | | | | | | | | | | | | | | | |  |
| 1. **In the past 12 months, how often did you drink alcoholic beverages?**   When we use the word “**drink**”, we are referring to either:   - one 341 ml or 12 oz serving of beer whether from a bottle, can, or draft - one 142 ml or 5 oz glass of wine or bottle of cooler - one straight or mixed drink with 1.5 oz (43ml) of liquor or spirit | | | | | | | | | | | | | | | | | | | | | | | | | | | | | | | | | | | | | | | | | | | | | | | | | | | | | | | | | | | | | | | |  |
|  | | ⃝ Never – **continue to Question 10** | | | | | | | | | | | | | | | | | | | | | | | | | | | | | | | | | | | | | | | | | | | | | | | | | | | | | | | | | | | | | |  |
|  | | ⃝ Less than once per month | | | | | | | | | | | | | | | | | | | | | | | | | | | | | | | | | | | | | | | | | | | | | | | | | | | | | | | | | | | | | |  |
|  | | ⃝ Once per month | | | | | | | | ⃝ 2-3 times per month | | | | | | | | | | | | | | | | | | | | | | | | | | | | | | | | | | | |  | | | | | | | | | | | | | | | | | |  |
|  | | ⃝ Once per week | | | | | | | | ⃝ 2-3 times per week | | | | | | | | | | | | | | | | | | | | | | | | | | | | | | | | | | | | ⃝ 4-5 times per week | | | | | | | | | | | | | | | | | |  |
|  | | ⃝ Daily or almost daily | | | | | | | | | | | | | | | | | | | | | | | | | | | | | | | | | | | | | | | | |  | | | | | | | | | | | | | | | | |  | | | |  |
| 1. **In the past 12 months, how many drinks did you usually have (on the days that you drank alcohol)?** | | | | | | | | | | | | | | | | | | | | | | | | | | | | | | | | | | | | | | | | | | | | | | | | | | | | | | | | | | | | | | | |  |
|  | | _________ drinks | | | | | | | | | | | | | | |  | | | | | | | | | | | | | | | | | | | | | | | | | |  | | | | | | | | | | | | | | |  | | | | | |  |
| 1. **Currently, do you smoke cigarettes?** | | | | | | | | | | | | | | | | | | | | | | | | | | | | | | | | | | | | | | | | | | | | | | | | | | | | | | | | | | | | | | | |  |
|  | | ⃝ No – continue to **Section 2** | | | | | | | | | | | | | | | | | | | | | | | | | |  | | | | | | | | | | | | | | | | | | | | | | | |  | | | | | | | | | | | |  |
|  | | ⃝ Occasionally | | | | | | | | | | | | | | | | | | | | | | | | | | ⃝ Every day | | | | | | | | | | | | | | | | | | | | | | | | | | | | | | | | | | | |  |
| 1. **On average, how many cigarettes do you smoke per day?** | | | | | | | | | | | | | | | | | | | | | | | | | | | | | | | | | | | | | | | | | | | | | | | | | | | | | | | | | | | | | | | |  |
|  | | __________ cigarettes | | | | | | | | | | | | | | |  | | | | | | | | | | | | | | | | | | | | | | | | | |  | | | | | | | | | | | | | | |  | | | | | |  |
| **Section 2: Cannabis Use**  Cannabis refers to ANY PRODUCTS derived from the cannabis plant (for example: dried leaves, oils, hashish, and edible products).  Other names for cannabis are marijuana, weed, pot, bud, and hash. | | | | | | | | | | | | | | | | | | | | | | | | | | | | | | | | | | | | | | | | | | | | | | | | | | | | | | | | | | | | | | |  |  |
| 1. **Have you EVER used or tried cannabis (or other cannabis related products)?** | | | | | | | | | | | | | | | | | | | | | | | | | | | | | | | | | | | | | | | | | | | | | | | | | | | | | | | | | | | | | | |  |  |
|  | ⃝ Yes  At what age did you first try cannabis? ____________ years old | | | | | | | | | | | | | | | | | | | | | | | | | | | | | | | | | | | | | | | | | | | | | | | | | | | | | | | | | |  | | | |  |  |
|  | ⃝ No – please go to **Question 27 (on Page 4)** | | | | | | | | | | | | | | | | | | | | | | | | | | | | | | | | | | | | | | | | | | | | | | | | | | | | | | | | | |  | | | |  |  |
| 1. **Have you used cannabis in the LAST 3 MONTHS?** | | | | | | | | | | | | | | | | | | | | | | | | | | | | | | | | | | | | | | | | | | | | | | | | | | | | | | | | | | | | | | |  |  |
|  | ⃝ Yes | | | | | | | | | | | | | | | | | | | | | | ⃝ No – please go to **Question 27 (on Page 4)** | | | | | | | | | | | | | | | | | | | | | | | | | | | | | | | | | | | | | | | |  |  |
| 1. **Which of the following best describes your cannabis use in the LAST 3 MONTHS?** | | | | | | | | | | | | | | | | | | | | | | | | | | | | | | | | | | | | | | | | | | | | | | | | | | | | | | | | | | | | | | |  |  |
|  | ⃝ Once or twice (in the last 3 months) | | | | | | | | | | | | | | | | | | | | | | | | | | | | | ⃝ Monthly (1-3 times per month) | | | | | | | | | | | | | | | | | | | | | | | | | | | | | | | | |  |  |
|  | ⃝ Weekly (1-4 times per week) | | | | | | | | | | | | | | | | | | | | | | | | | | | | ⃝ Daily or almost daily (5-7 days per week) | | | | | | | | | | | | | | | | | | | | | | | | | | | | | | | | | |  |  |
|  | ⃝ More than once per day | | | | | | | | | | | | | | | | | | | | | | | | | | | | | | | | | | | | | | | | | | | | | | | | | | | | | | | | | | | | | |  |  |
| 1. **On average, how much cannabis do you use per occasion/sitting?** | | | | | | | | | | | | | | | | | | | | | | | | | | | | | | | | | | | | | | | | | | | | | | | | | | | | | | | | | | | | | | |  |  |
|  | ⃝ Less than 0.5 grams | | | | | | | | | | | ⃝ 0.6 to 1 gram | | | | | | | | | | | | | | | | | | | | | | | | | ⃝ 1.1 to 2 grams | | | | | | | | | | | | | | | | | | | ⃝ 2.1 to 3 grams | | | | | | |  |  |
|  | ⃝ More than 3 grams | | | | | | | | | | ⃝ Not sure | | | | | | | | | | | | | | | | | | | | | | | | | | |  | | | | | |  | | | | | | | | |  | | | | | |  | | | |  |  |
|  | ⃝ Other – specify: _________________________________ | | | | | | | | | | | | | | | | | | | | | | | | | | | | | | | | | | | | | | | | | | | | | | | | | | | | | | | | | | | | | |  |  |
| 1. **What type(s) of cannabis product do you use? (select all that apply)** | | | | | | | | | | | | | | | | | | | | | | | | | | | | | | | | | | | | | | | | | | | | | | | | | | | | | | | | | | | | | | |  |  |
|  | ☐ Dried flower | | | | | | | | | | | | | | | | | | | | ☐ Cannabis vaporizer | | | | | | | | | | | | | | | | | | | | | | | | | | | | | | | | | | | | | | | | | |  |  |
|  | ☐ Liquid concentrate (eg. oil) | | | | | | | | | | | | | | | | | | | | ☐ Liquid (eg. Cola, tea) | | | | | | | | | | | | | | | | | | | | | | | | | | | | | | | | | | | | | | | | | |  |  |
|  | ☐ Capsule | | | | | | | | | | | | | | | | | | | | ☐ Edible products | | | | | | | | | | | | | | | | | | | | | | | | | | | | | | | | | | | | | | | | | |  |  |
|  | ☐ Hashish/kief | | | | | | | | | | | | | | | | | | | | ☐ Solid concentrate (eg. Shatter, budder) | | | | | | | | | | | | | | | | | | | | | | | | | | | | | | | | | | | | | | | | | |  |  |
|  | ☐ Other – specify: __________________________________ | | | | | | | | | | | | | | | | | | | | | | | | | | | | | | | | | | | | | | | | | | | | | | | | | | | | | | | | | | | | | |  |  |
|  | | | | | | | | | | | | | | | | | | | | | | | | | | | | | | | | | | | | | | | | | | | | | | | | | | | | | | | | | | | | | | |  |  |
| 1. **For the cannabis product that you MOST OFTEN use, what are the relative THC and CBD amounts?** | | | | | | | | | | | | | | | | | | | | | | | | | | | | | | | | | | | | | | | | | | | | | | | | | | | | | | | | | | | | | | |  |  |
|  | ⃝ Equal | | | ⃝ **More THC** than CBD | | | | | | | | | | | | | | | | | | | | | | | ⃝ **More CBD** than THC | | | | | | | | | | | | | | | | | | | | | | ⃝ Not sure | | | | | | | | | | | | | |  |  |
| 1. **What route do you currently use cannabis? (select all that apply)** | | | | | | | | | | | | | | | | | | | | | | | | | | | | | | | | | | | | | | | | | | | | | | | | | | | | | | | | | | | | | | |  |  |
|  | ☐ Smoke | | | | | ☐ Vape | | | | | | | | | | | | | | | | | ☐ Eat by mouth | | | | | | | | | | | | | | | | | ☐ Under the tongue | | | | | | | | | | | | | | | | | | | | | | |  |  |
|  | ☐ Apply to skin | | | | | ☐ Rectal | | | | | | | | | | | | | | | | | ☐ Other – specify: _______________ | | | | | | | | | | | | | | | | | | | | | | | | | | | | | | | | | | | | | | | |  |  |
| 1. **In the LAST 3 MONTHS, where did you get the cannabis you used? (select all that apply)** | | | | | | | | | | | | | | | | | | | | | | | | | | | | | | | | | | | | | | | | | | | | | | | | | | | | | | | | | | | | | | |  |  |
|  | ☐ I grow it myself | | | | | | | | | | | | | | | | | | ☐ Family or friends | | | | | | | | | | | | | | | | | | | | | | | | | | | | ☐ Dealer | | | | | | | | | | | | | | | |  |  |
|  | ☐ Storefront or dispensary | | | | | | | | | | | | | | | | | | ☐ Authorized retail store | | | | | | | | | | | | | | | | | | | | | | | | | | | |  | | | | | | | | | | | | | | | |  |  |
|  | ☐ Online licensed producer | | | | | | | | | | | | | | | | | | ☐ Online from other source | | | | | | | | | | | | | | | | | | | | | | | | | | | | | | | | | | | | | | | | | | | |  |  |
|  | ☐ Other – specify: _______________ | | | | | | | | | | | | | | | | | | | | | | | | | | | | | | | | | | | | | | | | | | | | | | | | | | | | | | | | | | | | | |  |  |
| 1. **On average, how much money do you spend on cannabis per MONTH?** | | | | | | | | | | | | | | | | | | | | | | | | | | | | | | | | | | | | | | | | | | | | | | | | | | | | | | | | | | | | | | |  |  |
|  | $ _________ | | | | | | | | | | | | | | | | | | | | | |  | | | | | | | | | | | | | | |  | | | | | | | | | | | | | | | | | | | | |  | | | |  |  |
| 1. **Why do you use cannabis? (select all that apply)** | | | | | | | | | | | | | | | | | | | | | | | | | | | | | | | | | | | | | | | | | | | | | | | | | | | | | | | | | | | | | | |  |  |
|  | ☐ For medical use (**with** a medical document) | | | | | | | | | | | | | | | | | | | | | | | | | | | | | | | | | | | | | | ☐ For recreational use | | | | | | | | | | | | | | | | | | | | | | | |  |  |
|  | ☐ For medical use (**without** a medical document) | | | | | | | | | | | | | | | | | | | | | | | | | | | | | | | | | | | | | |  | | | | | | | | | | | | | | | | | | | |  | | | |  |  |
| 1. **What is the MAIN REASON you use cannabis? (select ONE)** | | | | | | | | | | | | | | | | | | | | | | | | | | | | | | | | | | | | | | | | | | | | | | | | | | | | | | | | | | | | | | |  |  |
|  | ⃝ For my nerve pain | | | | | | | | | | | | | | ⃝ For other types of pain | | | | | | | | | | | | | | | | | | | | | | | | | | | | | | ⃝ To help me sleep | | | | | | | | | | | | | | | | | |  |  |
|  | ⃝ To improve my anxiety | | | | | | | | | | | | | | ⃝ To improve my mood | | | | | | | | | | | | | | | | | | | | | | | | | | | | | | ⃝ To increase my appetite | | | | | | | | | | | | | | | | | |  |  |
|  | ⃝ For recreational use | | | | | | | | | | | | | | ⃝ Other – specify: ____________________________________ | | | | | | | | | | | | | | | | | | | | | | | | | | | | | | | | | | | | | | | | | | | | | | | |  |  |
| 1. **Are there OTHER REASONS that you use cannabis? If yes, select all that apply.** | | | | | | | | | | | | | | | | | | | | | | | | | | | | | | | | | | | | | | | | | | | | | | | | | | | | | | | | | | | | | | |  |  |
|  | ☐ Not applicable (I only use cannabis for the reason above) | | | | | | | | | | | | | | | | | | | | | | | | | | | | | | | | | | | | | | | | | | | | | | | | | | | | | | | | | | | | | |  |  |
|  | ☐ For my nerve pain | | | | | | | | | | | | | ☐ For other types of pain | | | | | | | | | | | | | | | | | | | | | | | | | | | | | | | ☐ To help me sleep | | | | | | | | | | | | | | | | | |  |  |
|  | ☐ To improve my anxiety | | | | | | | | | | | | | ☐ To improve my mood | | | | | | | | | | | | | | | | | | | | | | | | | | | | | | | ☐ To increase my appetite | | | | | | | | | | | | | | | | | |  |  |
|  | ☐ For recreational use | | | | | | | | | | | | | ☐ Other – specify: _____________________________________ | | | | | | | | | | | | | | | | | | | | | | | | | | | | | | | | | | | | | | | | | | | | | | | | |  |  |
| 1. **Do you feel that cannabis improves your symptoms? If so, which one?** | | | | | | | | | | | | | | | | | | | | | | | | | | | | | | | | | | | | | | | | | | | | | | | | | | | | | | | | | | | | | | |  |  |
|  | ⃝ Yes  It helps with: _________________________________________________________ | | | | | | | | | | | | | | | | | | | | | | | | | | | | | | | | | | | | | | | | | | | | | | | | | | | | | | | | | | | | | |  |  |
|  | ⃝ No, it is not effective for me. | | | | | | | | | | | | | | | | | | | | | | | | | | | | | | | | | | | | | | | | | | | | | | | | | | | | | | | | | | | | | |  |  |
| 1. **What do you think are benefits of using cannabis (instead of other medications)? (select all that apply)** | | | | | | | | | | | | | | | | | | | | | | | | | | | | | | | | | | | | | | | | | | | | | | | | | | | | | | | | | | | | | | |  |  |
|  | ☐ It comes from a natural plant | | | | | | | | | | | | | | | | | | | | | | | | | | | | | | | | | | ☐ It is safer than other medications | | | | | | | | | | | | | | | | | | | | | | | | | | | |  |  |
|  | ☐ It is more effective than other medications | | | | | | | | | | | | | | | | | | | | | | | | | | | | | | | | | | ☐ It is easy to buy | | | | | | | | | | | | | | | | | | | | | | | | | | | |  |  |
|  | ☐ My healthcare provider recommends it | | | | | | | | | | | | | | | | | | | | | | | | | | | | | | | | | | ☐ My family and friends recommend it | | | | | | | | | | | | | | | | | | | | | | | | | | | |  |  |
|  | ☐ Other – specify: ______________________________________________________________ | | | | | | | | | | | | | | | | | | | | | | | | | | | | | | | | | | | | | | | | | | | | | | | | | | | | | | | | | | | | | |  |  |
| 1. **Do you have any concerns about using cannabis? (select all that apply)** | | | | | | | | | | | | | | | | | | | | | | | | | | | | | | | | | | | | | | | | | | | | | | | | | | | | | | | | | | | | | | |  |  |
|  | ☐ Side effects | | | | | | | | | | | | | | | | | | | | | | | | | | | | | | ☐ Effects on my ability to drive | | | | | | | | | | | | | | | | | | | | | | | | | | | | | | | |  |  |
|  | ☐ Fear of addiction | | | | | | | | | | | | | | | | | | | | | | | | | | | | | | ☐ Effects on my concentration | | | | | | | | | | | | | | | | | | | | | | | | | | | | | | | |  |  |
|  | ☐ Interactions with other medications | | | | | | | | | | | | | | | | | | | | | | | | | | | | | | ☐ Frequent dosing schedule | | | | | | | | | | | | | | | | | | | | | | | | | | | | | | | |  |  |
|  | ☐ Difficult to access/buy | | | | | | | | | | | | | | | | | | | | | | | | | | | | | | ☐ The high price | | | | | | | | | | | | | | | | | | | | | | | | | | | | | | | |  |  |
|  | ☐ Fear of negative judgement from others | | | | | | | | | | | | | | | | | | | | | | | | | | | | | | ☐ Effects on my family/personal relationships | | | | | | | | | | | | | | | | | | | | | | | | | | | | | | | |  |  |
|  | ☐ None of the above | | | | | | | | | | | | | | | | | | | | | | | | | | | | | | ☐ Other – specify: _________________________ | | | | | | | | | | | | | | | | | | | | | | | | | | | | | | | |  |  |
| **Please move to Section 3** | | | | | | | | | | | | | | | | | | | | | | | | | | | | | | | | | | | | | | | | | | | | | | | | | | | | | | | | | | | | | | |  |  |
| 1. **Have you ever considered using cannabis?** | | | | | | | | | | | | | | | | | | | | | | | | | | | | | | | | | | | | | | | | | | | | | | | | | | | | | | | | | | | | | | |  |  |
|  | ⃝ Yes (for **medical** use) | | | | | | | | | | | | | | | | | | | | | ⃝ No – move to **Question 29 (Section 3)** | | | | | | | | | | | | | | | | | | | | | | | | | | | | | | | | | | | | | | | | |  |  |
|  | ⃝ Yes (for **recreational** use) | | | | | | | | | | | | | | | | | | | | | | | | | | | | | | | | | | | | |  | | | | | | | | | | | | | | | | | | | | | | | | |  |  |
| 1. **If you answered YES, what are reasons you did NOT start using cannabis? (select all that apply)** | | | | | | | | | | | | | | | | | | | | | | | | | | | | | | | | | | | | | | | | | | | | | | | | | | | | | | | | | | | | | | |  |  |
|  | ☐ Side effects | | | | | | | | | | | | | | | | | | | | | | | | | | | | | | | ☐ Effects on my ability to drive | | | | | | | | | | | | | | | | | | | | | | | | | | | | | | |  |  |
|  | ☐ Fear of addiction | | | | | | | | | | | | | | | | | | | | | | | | | | | | | | | ☐ Effects on my concentration | | | | | | | | | | | | | | | | | | | | | | | | | | | | | | |  |  |
|  | ☐ Interactions with other medications | | | | | | | | | | | | | | | | | | | | | | | | | | | | | | | ☐ Frequent dosing schedule | | | | | | | | | | | | | | | | | | | | | | | | | | | | | | |  |  |
|  | ☐ Difficult to access/buy | | | | | | | | | | | | | | | | | | | | | | | | | | | | | | | ☐ The high price | | | | | | | | | | | | | | | | | | | | | | | | | | | | | | |  |  |
|  | ☐ Fear of negative judgement from others | | | | | | | | | | | | | | | | | | | | | | | | | | | | | | | ☐ Effects on my family/personal relationships | | | | | | | | | | | | | | | | | | | | | | | | | | | | | | |  |  |
|  | ☐ Doctor or other healthcare professional did not recommend it | | | | | | | | | | | | | | | | | | | | | | | | | | | | | | | | | | | | | | | | | | | | | | | | | | | | | | | | | | | | | |  |  |
|  | ☐ None of the above | | | | | | | | | | | | | | | | | | | | | | | | | | | | | | |  | | | | | | | | | | | | | | | | | | | | | | | | | | | | | | |  |  |
|  | ☐ Other – specify: ____________________________________________________________ | | | | | | | | | | | | | | | | | | | | | | | | | | | | | | | | | | | | | | | | | | | | | | | | | | | | | | | | | | | | | |  |  |
| **Section 3: Cannabis and Hemodialysis** | | | | | | | | | | | | | | | | | | | | | | | | | | | | | | | | | | | | | | | | | | | | | | | | | | | | | | | | | | | | | | |  |  |
| 1. **Has any member of the hemodialysis team asked whether you have used cannabis?**   **(apart from this survey)** | | | | | | | | | | | | | | | | | | | | | | | | | | | | | | | | | | | | | | | | | | | | | | | | | | | | | | | | | | | | | | |  |  |
|  | | | ⃝ Yes | | | | ⃝ No | | | | | | | | | | | | | | | | | |  | | | | | | | | | | | | | | |  | | | | | | | | | | | | | | | | |  | | | | | |  |  |
| 1. **Have YOU ever asked a member of the hemodialysis team about cannabis?** | | | | | | | | | | | | | | | | | | | | | | | | | | | | | | | | | | | | | | | | | | | | | | | | | | | | | | | | | | | | | | |  |  |
|  | | | ⃝ Yes | | | | ⃝ No | | | | | | | | | | | | | | | | | |  | | | | | | | | | | | | | | |  | | | | | | | | | | | | | | | | |  | | | | | |  |  |
| 1. **What source of information about cannabis do you think is the MOST reliable?** | | | | | | | | | | | | | | | | | | | | | | | | | | | | | | | | | | | | | | | | | | | | | | | | | | | | | | | | | | | | | | |  |  |
|  | | | ⃝ My family and friends | | | | | | | | | | | | | ⃝ Articles I find on the Internet | | | | | | | | | | | | | | | | | | | | | | | | | | | | | | | |  | | | | | | | | | | | | | | |  |  |
|  | | | ⃝ My family physician | | | | | | | | | | | | | ⃝ My hemodialysis team | | | | | | | | | | | | | | | | | | | | | | | | | | | | | | | | ⃝ My community pharmacist | | | | | | | | | | | | | | |  |  |
|  | | | ⃝ Cannabis retailers | | | | | | | | | | | | | ⃝ Medical cannabis clinic | | | | | | | | | | | | | | | | | | | | | | | | | | | | | | | | | | | | | | | | | | | | | | |  |  |
|  | | | ⃝ Other – specify: _______________________________________________ | | | | | | | | | | | | | | | | | | | | | | | | | | | | | | | | | | | | | | | | | | | | | | | | | | | | | | | | | | | |  |  |
| 1. **What type of information about cannabis do you want to know the most about?** | | | | | | | | | | | | | | | | | | | | | | | | | | | | | | | | | | | | | | | | | | | | | | | | | | | | | | | | | | | | | | |  |  |
|  | | | ⃝ What I can use it for | | | | | | | | | | | | | | | | | ⃝ How to pick the right product for me | | | | | | | | | | | | | | | | | | | | | | | | | | | | | | | | | | | | | | | | | | |  |  |
|  | | | ⃝ How much to use | | | | | | | | | | | | | | | | | ⃝ Where to buy cannabis | | | | | | | | | | | | | | | | | | | | | | | | | | | | | | | | | | | | | | | | | | |  |  |
|  | | | ⃝ Side effects | | | | | | | | | | | | | | | | | ⃝ If it interacts with my other medications | | | | | | | | | | | | | | | | | | | | | | | | | | | | | | | | | | | | | | | | | | |  |  |
|  | | | ⃝ Other – specify: _______________________________________________ | | | | | | | | | | | | | | | | | | | | | | | | | | | | | | | | | | | | | | | | | | | | | | | | | | | | | | | | | | | |  |  |
| 1. **Rate the following statements:** | | | | | | | | | | | | | | | | | | | | | | | | | | | | | | | | | | | | | | | | | | | | | | | | | | | | | | | | | | | | | | | | |
|  | | | | | | | | | | | | | | | | | | | | | | | | **Strongly agree** | | | | | | | | | | | | **Agree** | | | | | | **Not sure** | | | | | | | | | | | | **Disagree** | | | | | | | **Strongly disagree** | | | |
| (a) I am comfortable talking to my hemodialysis team about using cannabis. | | | | | | | | | | | | | | | | | | | | | | | | ⃝ | | | | | | | | | | | | ⃝ | | | | | | ⃝ | | | | | | | | | | | | ⃝ | | | | | | | ⃝ | | | |
| (b) My hemodialysis team would support me if I wanted to use cannabis. | | | | | | | | | | | | | | | | | | | | | | | | ⃝ | | | | | | | | | | | | ⃝ | | | | | | ⃝ | | | | | | | | | | | | ⃝ | | | | | | | ⃝ | | | |
| (c) My hemodialysis team is knowledgeable about cannabis and its effects on my health. | | | | | | | | | | | | | | | | | | | | | | | | ⃝ | | | | | | | | | | | | ⃝ | | | | | | ⃝ | | | | | | | | | | | | ⃝ | | | | | | | ⃝ | | | |
| (d) Cannabis retailers are knowledgeable about cannabis and its effects on my health. | | | | | | | | | | | | | | | | | | | | | | | | ⃝ | | | | | | | | | | | | ⃝ | | | | | | ⃝ | | | | | | | | | | | | ⃝ | | | | | | | ⃝ | | | |
| (e) Cannabis has beneficial health effects. | | | | | | | | | | | | | | | | | | | | | | | | ⃝ | | | | | | | | | | | | ⃝ | | | | | | ⃝ | | | | | | | | | | | | ⃝ | | | | | | | ⃝ | | | |
| (f) Cannabis has harmful health effects. | | | | | | | | | | | | | | | | | | | | | | | | ⃝ | | | | | | | | | | | | ⃝ | | | | | | ⃝ | | | | | | | | | | | | ⃝ | | | | | | | ⃝ | | | |
| (g) Cannabis is harmful to my hemodialysis treatment. | | | | | | | | | | | | | | | | | | | | | | | | ⃝ | | | | | | | | | | | | ⃝ | | | | | | ⃝ | | | | | | | | | | | | ⃝ | | | | | | | ⃝ | | | |
| **END OF SURVEY – Please return to the Survey Collection Box at the entrance of the hemodialysis units.** | | | | | | | | | | | | | | | | | | | | | | | | | | | | | | | | | | | | | | | | | | | | | | | | | | | | | | | | | | | | | | | | |
